# Objectively measured sedentary behaviour and moderate and vigorous physical activity in different school subjects: a cross-sectional study

**DOI:** 10.1186/s12889-017-4046-9

**Published:** 2017-01-23

**Authors:** Kerli Mooses, Katrin Mägi, Eva-Maria Riso, Maarja Kalma, Priit Kaasik, Merike Kull

**Affiliations:** Institute of Sport Sciences and Physiotherapy, University of Tartu, Tartu, Estonia

**Keywords:** MVPA, Sedentary, Sedentary bout, Lesson time, Physical activity

## Abstract

**Background:**

Evidence shows the positive influence of moderate and vigorous physical activity (MVPA) and negative influence of sedentary time on health and academic achievement. Although schools can significantly contribute to overall physical activity, little is known about MVPA and sedentary behaviour in different school subjects in different grades.

**Methods:**

Physical activity of 646 students from 18 schools (94 classes) and from three school stages (grades 1–9, aged 7–16) was measured with accelerometry for 5 school days. Time and proportion of MVPA and sedentary time, also average sedentary bout length was calculated for native language (Estonian), mathematics, science, foreign language, music and crafts lessons.

**Results:**

A total of 6363 lessons were measured, with lesson duration of 45 min. The average lesson time MVPA remained below 2.2 min in all school stages and in all subjects. Students in grades 4–6 had greatest decline in the proportion of lesson time MVPA in science (β = −1.9, 95%CI −3.1– -0.6) and music (−1.2, −2.1– -0.4) and in grades 7–9 in music (−1.7, −3.1– -0.3) lessons compared to grades 1–3. In grades 1–3 students spent on average 76% of lesson time (34.0 ± 7.0 min) as sedentary, whereas in grades 7–9 the average proportion of sedentary time was 87% (38.9 ± 5.7 min). An average sedentary bout length increased from 13 min in grades 1–3 to 20 min in grades 7–9. An increase in sedentary bout length from grades 1–3 compared to grades 7–9 was present in most subjects, except crafts, with smallest increase in foreign language (6 min, 3.5–8.9) and greatest in music lessons (16.6 min, 11.9–21.3). Lessons with prolonged sedentary bouts formed a maximum 36% of all lessons in grades 1–3 and 73% in grades 7–9.

**Conclusion:**

The long sedentary time, bout length and low MVPA in most subjects were unfavourable in respect of both health and academic achievement. Significantly increasing sedentary time and sedentary bout length in older school stages highlights the need for interventions in all subjects and especially in older grades in order to combat the inactivity of children.

## Background

Physical activity is one of the most important factors in obesity and disease prevention [1, 2] with a dose–response relationship indicating that more physical activity is associated with additional health benefits [1, 3]. There is also a growing body of evidence highlighting the importance of physical activity and physical fitness on cognition [4], on-task behaviour [5–7] and academic achievement [8–10]. It has been advised that children should accumulate a minimum of 60 min of moderate and vigorous physical activity (MVPA) every day [3]. At the same time, sedentary behaviour has been identified as an independent health risk factor [11, 12] and associated with increased mortality risk in adults [13, 14]. As for children, sedentary behaviour has been shown to be related with greater increase in body mass index (BMI) [11] and other cardio-metabolic risk factors [15, 16]. Therefore, it is recommended to break up sitting as frequently as possible and avoid prolonged sitting [12, 17]. Despite all the recommendations, the physical activity of European children remain low [18] and increasing the physical activity levels and decreasing sedentary time of children remains a great challenge in public health.

Based on the accumulated experience from implementing physical activity interventions, the partnership outside the health sector has been pointed out [19]. Therefore, whole-school approach which focuses on physical education lessons, enabling physical activity during recess and academic lessons and engaging school staff, students and home, have been targeted as one of the seven best investments in increasing children daily physical activity levels and reducing sedentary time [20]. Another advantage of school setting is the opportunity to reach children with different socio-economic background. It has been advised that students should accumulate at least 50% of daily MVPA at school [21], but according to studies in-school MVPA accounts for 22–40% of daily MVPA [22–25]. As majority of in-school time is spent in lessons and movement in the classroom has been associated with improved on-task behaviour [6], academic achievement [8–10], increased physical activity and decreased sedentary levels [26], lesson time is an important segment to be addressed in school setting. Nevertheless, according to recent studies, the mean proportion on MVPA during academic lessons is approximately 1% [24, 26], while others have found that MVPA can make up to 13% of lesson time [27, 28]. In addition, more than 70% of lesson time is spent as sedentary [26–28]. However, to date previous research [24, 26–28] have described all subjects as overall lesson time and there has been no attempt for detailed analysis of the physical activity and sedentary behaviour in difference subjects. Furthermore, a number of studies recognize the importance of interrupting the prolonged sedentary behaviour [15, 29]. Nonetheless, there is only some research showing students having more uninterrupted sedentary time at school compared to non-school time [30] and to the best of our knowledge there is no research describing the sedentary bouts either during lesson time or in different subjects.

There is an ample of evidence that overall physical activity levels reduce and sedentary time increase with age [18, 31, 32]. In order to understand better the contributing factors for physical inactivity in age group 13–18, when the decline in physical activity is the greatest [31], it is important to know how different in-school segments (academic lessons, recess, PE etc.) can support the physical activity of students. However, there are only few studies investigating the MVPA and sedentary behaviour during lesson time in different grades [33, 34]. Brooke et al. [34] recently showed with a longitudinal study that between ages 10 to 14 the total physical activity during lesson time declined whereas MVPA remained the same. Similar tendency is found on school level where total amount of physical activity acquired in school hours is reduced during transition from primary school to secondary school [22]. Nonetheless, there current literature has not described physical activity and sedentary time in different subjects and how the activity in different subjects change with age, which is an important input when planning interventions. Therefore, this paper aims to fill a gap in current literature and give an overview of physical activity, sedentary time and sedentary behaviour in different subject and in different school stages. The aims were to (i) describe objectively measured MVPA, sedentary time and prolonged sequences of sedentary time (sedentary bouts) and, (ii) investigate the changes in MVPA, sedentary time and sedentary bouts throughout the ages 7–16 in different school subjects.

## Methods

The invitation to participate in the cross-sectional study was sent to randomly selected comprehensive schools from all over Estonia. Special schools for students with mental and/or physical disability were excluded from the sample. After receiving consent from school, students attending basic school (children aged 7–16) were asked to participate in the study. Signed written informed consent was obtained from 1756 parents and their children resulting in a 62.4% participation rate. From consented children a randomly selected subsample was formed to carry out the study so that all consented schools and classes were represented. As a result, physical activity was measured in 18 schools and 756 students from 94 classes. From 756 students measured, 110 were excluded from the analysis due to accelerometer malfunction or not meeting the wear time criteria, leaving 646 students into the final analysis. Excluded children did not differ from those entered into the analysis in terms of gender distribution and BMI (*p* = 0.643, *p* = 0.685 respectively). The study was approved by the Research Ethics Committee of the University of Tartu (nr 242 T-17 and 255/M-11).

The physical activity of students was measured for one school week during the period of December 2014 to April 2016, where grades 1–6 were measured from December 2014 to May 2015 and grades 7–9 from January 2016 to April 2016 with accelerometer Actigraph GT3X (ActiGraph LLC, Penascola, FL, USA) using 15-s epochs. On the first day of measurement students were instructed on how to attach the accelerometer on the hip and to fill the accelerometer diary. Students were advised to remove the accelerometer for water based activities (e.g. showering, swimming etc.) and retain their usual activity. After the distribution of accelerometers, selected demographic (age, gender) and anthropometric variables were measured at school by trained researchers. Height (Seca 213, Seca GmbH, Germany) and body mass (A&D Instruments, Abington, UK) were measured to the nearest 0.1 cm and 0.1 kg respectively and body mass index (BMI) was calculated. Age-specific BMI cut-off points were used to classify students as normal or overweight/obese according to International Obesity Task Force [35].

Collected data was downloaded and processed using ActiLife software (v 6.13.2, ActiGraph LLC, Penascola, FL, USA). Data was considered valid if a minimum of 240 min of wear time was present [7, 26]. Consecutive zero counts of 60 min were classified as non-wear time [7]. The data of first measurement day was excluded in order to eliminate potential bias caused by the distribution of accelerometers and performing anthropometric measurements during lessons. Time filters, assigned based on school time-tables and the diaries filled by students, were applied to get the physical activity data of different subjects. School subjects included into the analysis were native language (Estonian), mathematics, science, foreign language, music and crafts/arts (hereinafter crafts) as these lessons were taught throughout the ages 7–16 in all participating schools. Physical education lessons were excluded from current analysis. In all participating schools a usual lesson lasted for 45 min being followed by a 10–15 min long break. To explore the differences between grades, the grades were aggregated according to stages of basic school, where stage 1 includes grades 1–3, stage 2 includes grades 4–6 and stage 3 includes grades 7–9.

Evenson cut-points were used to calculate minutes spent in sedentary (≤100 counts/minute), light (101–2295 counts/minute) and MVPA (≥2296 counts/minute) [36], which have shown best classification accuracy in children [37]. Time spent in MVPA and sedentary was presented both in minutes and as a proportion from lesson time. A sedentary bout was defined as a time when counts per minute were below 100. The bout stopped when the accelerometer counts per minute were ≥ 100 for 3 or more consecutive minutes [38]. The number of sedentary bouts lasting <15 min, 15–29.9 min, 30–39.9 min and 40–45 min were calculated for each participant in each lesson. Also an average bout length for each participant in each lesson was computed.

### Data analysis

Descriptive analysis was conducted to explore group differences using factorial ANOVAs with Tukey post-hoc test, for categorical data chi-square test was used. When more than 2 groups were present, several chi-square tests between all groups were performed and adjusted p-values calculated. The statistical significance was set at *p* < 0.05. In order to take into account the nested structure of the data, the differences between school stages were analysed using linear mixed models where lessons were nested in schools. Models were separately run for sedentary time, MVPA and average sedentary bout length as dependent variable for each subject. All models were controlled for BMI and gender. Data was analysed using statistical program R version 3.0.2 (http://www.r-project.org/), for linear mixed models the package lme4 was used [39]. The statistical significance of the model estimates was evaluated using 95% confidence intervals.

## Results

The characteristics of included students are presented in Table 1. Although the BMI between three school stages was significantly different, the proportion of overweight/obese students was approximately one third in all school stages.

**Table 1:** Characteristics of participants by school stages

	Grades 1–3 *N* = 305	Grades 4–6 *N* = 258	Grades 7–9 *N* = 83
Gender (%)			
Male	50.8	53.1	60.2
Female	49.2	46.9	39.8
Age (y)	7.9 ± 0.7	11.0 ± 0.8^*^	14.6 ± 1.1^*,#^
Height (m)	1.34 ± 0.07	1.51 ± 0.09^*^	1.69 ± 0.08^*,#^
Weight (kg)	32.0 ± 7.8	45.1 ± 12.5^*^	62.4 ± 13.1 ^*,#^
BMI (kg/m^2^)	17.6 ± 2.9	19.6 ± 4.0^*^	21.7 ± 3.4^*,#^
BMI z-score	−0.36 ± 0.78	0.17 ± 1.08^*^	0.74 ± 0.91^*,#^
Weight status (%)			
Under weight or normal	69.0	72.1	70.7
Overweight or obese	31.0	27.9	29.3

Data presented as mean ± standard deviation or %. BMI – body mass index. Overweight or obese – age-specific BMI corresponding to BMI ≥ 25.0 at age 18

*different from grades 1–3, *p* < 0.05

#different from grades 4–6, *p* < 0.05

A total of 6363 lessons were measured – 2529 lessons in grades 1–3, 2956 lessons in grades 4–6 and 878 lessons in grades 7–9 (Table 2).

**Table 2:** Number of lessons measured and physical activity and length of sedentary bouts by subject and school stage

	Grades 1–3	Grades 4–6	Grades 7–9
Number of lessons measured			
Native language	837	711	215
Mathematics	645	786	251
Science	205	333	41
Foreign language	134	607	242
Music	381	257	61
Crafts	327	262	68
Total	2529	2956	878
MVPA minutes			
Native language	0.5 ± 0.8 (1.0%)	0.2 ± 0.4 (0.6%)^*^	0.4 ± 0.5 (0.9%)
Mathematics	0.6 ± 1.1 (1.3%)	0.2 ± 0.5 (0.5%)^*^	0.2 ± 0.4 (0.5%)^*^
Science	1.1 ± 2.1 (2.4%)	0.2 ± 0.5 (0.5%)^*^	0.2 ± 0.3 (0.4%)
Foreign language	0.7 ± 1.0 (1.5%)	0.3 ± 0.6 (0.7%)^*^	0.7 ± 2.5 (1.6%)
Music	1.1 ± 1.4 (2.5%)	0.5 ± 0.7 (1.1%)^*^	0.2 ± 0.3 (0.5%)^*^
Crafts	0.7 ± 1.0 (1.6%)	0.7 ± 1.1 (1.6%)	2.2 ± 3.5 (4.8%)^*,#^
Total	0.7 ± 1.2 (1.5%)	0.3 ± 0.6 (0.7%)^*^	0.5 ± 1.8 (1.2%)^*,#^
Sedentary minutes			
Native language	35.8 ± 5.9 (79.5%)	38.5 ± 4.5 (85.6%)^*^	39.3 ± 5.0 (87.4%)^*^
Mathematics	34.5 ± 7.5 (76.8%)	38.9 ± 4.7 (86.4%)^*^	40.1 ± 4.5 (89.1%)^*^
Science	32.1 ± 8.9 (71.4%)	38.2 ± 5.1 (85.0%)^*^	38.9 ± 4.5 (86.3%)^*^
Foreign language	33.1 ± 7.3 (73.6%)	37.4 ± 5.6 (83.2%)^*^	39.0 ± 5.7 (86.7%)^*^
Music	31.6 ± 7.2 (70.2%)	35.5 ± 6.1 (78.9%)^*^	40.5 ± 3.9 (90.0%)^*,#^
Crafts	32.6 ± 5.8 (72.5%)	32.9 ± 6.0 (73.1%)	32.3 ± 8.1 (71.9%)
Total	34.0 ± 7.0 (75.5%)	37.6 ± 5.4 (83.6%)^*^	38.9 ± 5.7 (86.5%)^*,#^
Sedentary bout length in minutes			
Native language	12.3 ± 9.6 (27.4%)	17.9 ± 13.1 (39.8%)^*^	20.2 ± 13.8 (44.9%)^*^
Mathematics	15.3 ± 10.9 (34.0%)	21.6 ± 14.0 (48.0%)^*^	25.1 ± 15.3 (55.7%)^*^
Science	14.1 ± 10.8 (31.3%)	20.3 ± 13.5 (45.2%)^*^	22.2 ± 13.7 (49.4%)^*^
Foreign language	14.3 ± 10.0 (31.7%)	18.2 ± 12.9 (40.5%)^*^	18.5 ± 13.8 (41.2%)
Music	13.2 ± 8.8 (29.4%)	16.6 ± 11.6 (36.9%)^*^	28.1 ± 14.8 (62.5%)^*,#^
Crafts	6.9 ± 9.0 (21.2%)	9.0 ± 7.7 (19.9%)	9.8 ± 8.9 (21.8%)
Total	12.8 ± 9.7 (28.5%)	17.3 ± 13.1 (38.6%)^*^	19.9 ± 14.5 (44.2%)^*,#^

Data presented as mean ± standard deviation (mean % from lesson time)

*different from grades 1–3, *p* < 0.05

#different from grades 4–6, *p* < 0.05

*MVPA* moderate and vigorous physical activity

Overall, the MVPA minutes acquired during lesson time remained below 2.2 min in all school stages (Table 2). In grades 1–3, significantly more time in MVPA was spent in science and music lessons compared to other subjects (*p* < 0.05), the lowest MVPA minutes were in native language. In grades 4–6 and 7–9 more MVPA minutes were acquired in crafts lessons compared to other subjects (*p* < 0.05). The lowest MVPA minutes in grades 4–6 were in native language, mathematics and science lessons and in grades 7–9 in mathematics, science and music lessons.

When comparing school stages, students in grades 4–6 had greatest decline in the proportion of lesson time MVPA in science (β = −1.9, 95%CI −3.1– -0.6) and music lessons (−1.2, −2.1– -0.4) compared to grades 1–3 when controlled for BMI and gender (Table 3). In grades 7–9 smaller proportion of MVPA was acquired in music (−1.7, −3.1– -0.3) lessons compared to grades 1–3.

**Table 3:** Physical activity and sedentary bout length between school stages by subjects

	Grades 4–6 (Ref: grades 1–3)	Grades 7–9 (Ref: grades 1–3)
Proportion of MVPA		
Native language	−0.6 (−0.9– -0.2) ^*^	−0.4 (−1.0–0.3)
Mathematics	−0.7 (−1.1– -0.2) ^*^	−0.7 (−1.3– -0.0) ^*^
Science	−1.9 (−3.1– -0.6) ^*^	−2.1 (−5.0–0.8)
Foreign language	−0.9 (−1.7– -0.2) ^*^	−0.3 (−1.2–0.6)
Music	−1.2 (−2.1– -0.4) ^*^	−1.7 (−3.1– -0.3) ^*^
Crafts	−0.1 (−1.1–1.0)	2.4 (0.7–4.0) ^*^
Proportion of sedentary time		
Native language	6.0 (3.9–8.1) ^*^	8.5 (4.3–12.6) ^*^
Mathematics	8.5 (5.4–11.6) ^*^	10.8 (6.1–15.6) ^*^
Science	11.7 (6.4–17.0) ^*^	14.6 (2.4–26.9) ^*^
Foreign language	10.0 (6.2–13.9) ^*^	12.6 (8.0–17.2) ^*^
Music	9.0 (4.3–12.8) ^*^	18.3 (11.3–25.2) ^*^
Crafts	0.3 (−3.0–3.5)	−2.5 (−8.4–3.4)
Sedentary bout length		
Native language	7.5 (5.1–10.0) ^*^	10.4 (6.5–14.3) ^*^
Mathematics	7.8 (5.3–10.2) ^*^	10.9 (6.8–15.0) ^*^
Science	7.7 (4.3–11.0) ^*^	10.3 (2.5–18.2) ^*^
Foreign language	5.5 (1.4–9.5) ^*^	5.6 (0.8–10.4) ^*^
Music	6.2 (3.5–8.9) ^*^	16.6 (11.9–21.3) ^*^
Crafts	0.6 (−1.8–2.9)	2.9 (−1.7–7.4)

Models are controlled for BMI and gender

Data are presented as beta coefficients (95% confidence interval)

*MVPA* moderate and vigorous physical activity

^*^different from grades 1–3, *p* < 0.05

A significant increase in sedentary time during lessons from first school stage to third was present in most subjects, except crafts (Table 2). In grades 1–3 students spent on average 76% of lesson time (34.0 ± 7.0 min) as sedentary, whereas in grades 7–9 the average proportion of sedentary time was 87% (38.9 ± 5.7 min). There was significantly more sedentary time present in native language lessons in grades 1–3 and less sedentary time in music and crafts lessons in grades 4–6 compared to other subjects (*p* < 0.05). Crafts remained the least sedentary subject also in grades 7–9 (*p* < 0.05). In grades 4–6, the greatest increase in the proportion of sedentary time were in science (11.7, 6.4–17.0) and foreign language lessons (10.0, 6.2–13.9) when compared with grades 1–3 and controlled for BMI and gender (Table 3). As for grades 7–9 the greatest increase in the proportion of sedentary lesson time was present in music (18.3; 11.3–25.2) and science (14.6; 2.4–26.9) lessons when compared with grades 1–3. There was no significant difference in the proportion of sedentary time between grades 1–3 and older school stages in crafts lessons.

**Fig. 1:**
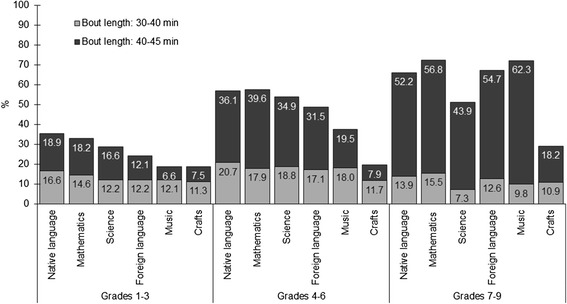
The proportion of lessons with prolonged sedentary bouts by subject and school stage

The average sedentary bout length was shortest in all school stages in craft lessons compared to other subjects (*p* < 0.05) remaining below 10 min (Table 2). In grades 1–3, the average sedentary bout length was longest in mathematics. In grades 4–6 average sedentary bout length in mathematics and science lessons was significantly longer compared to other subjects (*p* < 0.05). In grades 7–9, music lessons had significantly longer average sedentary bout length compared to most subjects (*p* < 0.05), except for mathematics. The analysis of sedentary bouts during lessons showed that an average sedentary bout length increased in all subjects, except in crafts, when comparing grades 1–3 with other school stages (Table 3), revealing a significant 16.6-min difference between grades 1–3 and grades 7–9 in music lessons when controlled for BMI and gender. In native language, mathematics, and science lessons an average bout length increased approximately 8 min in grades 4–6 and 10 min in grades 7–9 compared to grades 1–3. Figure 1 shows the increase in prolonged sedentary bouts by subjects. In grades 1–3 lessons with prolonged sedentary bouts formed a maximum 36% of all lessons and in grades 7–9 the proportion has increased to 73%.

## Discussion

The results of this study extends the current literature by giving a detailed information about physical activity levels in different subjects and school stages. We demonstrated that the sedentary behaviour throughout the basic school changed in unfavourable direction – the sedentary time, the average length of sedentary bouts and the proportion of prolonged sedentary bouts increased with school stages in most subjects. Another important finding was that the MVPA levels in different subjects were low and in most subjects the lesson time MVPA levels decreased with school stages. To the best of our knowledge present study is the first to describe physical activity, sedentary time and sedentary bouts during lesson time throughout the grades 1–9 in different subjects. The novel findings of current study highlight the need to take actions in school settings and in all subjects to combat the inactivity of children and help to aid decision-making when designing interventions.

Our study showed that a majority of lesson time was spent as sedentary in most subjects, ranging from 76% in grades 1–3 to 87% in grades 7–9, which is in line with previous findings [26–28]. However, previous research has concentrated on overall class time [27, 28] or certain subjects like mathematics and English [26], therefore our study adds new insight regarding sedentary time in different subjects. For example, the most sedentary subject in grades 1–3 was native language, whereas craft was the least sedentary subject in grades 4–6 and 7–9. Sedentary behaviour has been associated with unfavourable health outcomes in children [15, 16, 38, 40] and therefore, public health interventions in cooperation with schools should aim to find solutions to reduce sedentary time in lessons. As even light intensity physical activity has beneficial effect on the health of children [41, 42], reducing sedentary time in all lessons might be a promising mean in public health perspective. Another aspect of reducing sedentary time is that sedentary behaviour tracks during childhood and adolescence [43]. Moreover, according to a review [43], sedentary behaviour appears to track more consistently than physical activity. Therefore, reducing the sedentary time of students during lesson time has the potential to have sustained benefits that carry over to later life.

Another important finding of present study was the low lesson time MVPA in all subjects throughout grades 1–9, accounting only for 0.5–4.8% of lesson time. According to previous studies the overall lesson time MVPA can range from 1 to 13% [24, 26–28]. The findings of current study emphasize the need for interventions to increase lesson time physical activity in all lessons, despite the subject taught, throughout the grades 1–9 and especially in grades 4–6, where the amount of lesson time MVPA was the lowest, remaining below 0.7 min in all subjects. Several previous interventions conducted on students aged 8–13 years have shown that classroom physical activity breaks are promising mean to increase lesson time physical activity and reduce sedentary time [7, 26, 44–47]. A recent study with 8–9 year-olds demonstrated that approximately a quarter of mathematics and English lessons, where physical activity was integrated into the lesson, was spent in MVPA and only a third in sedentary [26]. A significant increase in MVPA and decrease in sedentary time during lessons with active breaks have been reported also by others [47, 48]. One important aspect of increasing lesson time MVPA is that each additional minute of school-day MVPA is associated with more than 1 additional minute of total daily MVPA [23]. Thus, activity breaks during lessons increase the likelihood of meeting the physical activity recommendations (60 min of MVPA every day [3]) by two-fold [7]. It can be concluded that increasing school and lesson time MVPA has important contribution in meeting the daily physical activity recommendations. The findings of our study present an important challenge to rearrange the traditional educational system where movement and physical activity would be a normality.

One of the issues emerging from current study is the reducing MVPA and increasing sedentary behaviour during lesson time in older school stages in most subjects. In our study, the proportion of sedentary time increased in all subjects, except crafts, throughout three school stages. For example, when compared to grades 1–3, the greatest increase in the proportion of sedentary time in grades 4–6 was in science and in grades 7–9 in music, while the smallest increase was in native language both in grades 4–6 and grades 7–9. Also, there was a significant increase in the average sedentary bout length. More precisely, depending on the subject, the average sedentary bout length increased 6–8 min in grades 4–6 and 5–17 min in grades 7–9 compared to grades 1–3. An increase in sedentary time during lessons has been found earlier [34], showing a decline in total physical activity during lesson time but not in MVPA between age 10–14 years, indicating that light physical activity is being replaced by sedentary behaviour. However, our finding concerning decline in the proportion of lesson time MVPA from grades 1–3 to 7–9 in several subjects contradicts the previous findings [34] and warrants further investigation. In addition, our study showed an increase in the proportion of lessons with prolonged (>30 min) sedentary bouts throughout the three school stages, forming up to 73% of all lessons in grades 7–9. Surprisingly, the highest increase in prolonged sedentary bouts was in music, where lessons with prolonged sedentary bout formed 18% of all music lessons in grades 1–3 compared to 72% in grades 7–9. It could be hypothesised that unfavourable changes in lesson time physical activity in most subjects were due to academic demands which also increase in older grades despite the research evidence showing association between lesson time physical activity breaks and better on-task behaviour [5–7, 47] or academic achievement [8–10]. It can be concluded, that physical activity is an unused resource during lesson time which supports both the academic goals as well as the overall health of students. Even the modest amounts of physical activity can have tremendous health benefits [1], especially as children do not compensate prolonged sedentary time in other parts of the day [49]. Therefore, more effort should be made to increase lesson time physical activity and reduce sedentary time despite the subject taught, with more attention paid on older grades. Whether the positive effects of activity breaks are present or which approach is feasible in older grades, warrants further investigation.

Interpreting the results of currents study, attention should be paid on the sample size in third school stage (grades 7–9), which is smaller than in other school stages, due to the malfunction of the accelerometers an approximately a quarter of data were lost. Small sample size resulted in larger confidence interval and therefore some significant differences between subjects or school stages could have remained undetected. However, present study is the first attempt in examining the MVPA and sedentary behaviour in older school stages (grades 7–9), thus filling the gap in the current literature. Also, despite acquiring time tables from schools and controlling them with students’ diaries, some misclassification of lessons could be present. Nevertheless, we believe this effect to be minimal.

## Conclusions

In summary, the evidence from this study shows that the lesson time physical activity is alarming as most lesson time was spent as sedentary, the proportion of lessons with prolonged sedentary bouts was considerable and time spent in MVPA was very low in all subjects. Moreover, the changes in physical activity from younger to older grades in most subjects were unfavourable in respect of both health and academic achievement. Our results highlight the need to reduce sedentary time in all subjects, with special attention on older grades. Present study gives a valuable input to planning future school-based interventions and supports political decisions aimed to reduce sedentary time in educational system.

## Abbreviations

BMI: Body mass index
MVPA: Moderate and vigorous physical activity.
